# Sustainable Valorization of Bioplastic Waste: A Review on Effective Recycling Routes for the Most Widely Used Biopolymers

**DOI:** 10.3390/ijms24097696

**Published:** 2023-04-22

**Authors:** Lorenzo Bartolucci, Stefano Cordiner, Emanuele De Maina, Gopalakrishnan Kumar, Pietro Mele, Vincenzo Mulone, Bartłomiej Igliński, Grzegorz Piechota

**Affiliations:** 1 Industrial Engineering Department, University of Rome Tor Vergata, Via del Politecnico 1, 00133 Rome, Italy; lorenzo.bartolucci@uniroma2.it (L.B.); cordiner@uniroma2.it (S.C.); emanuele.de.maina@uniroma2.it (E.D.M.); pietro.mele@uniroma2.it (P.M.); mulone@uniroma2.it (V.M.); 2 Institute of Chemistry, Bioscience and Environmental Engineering, Faculty of Science and Technology, University of Stavanger, 4036 Stavanger, Norway; gopalakrishnanchml@gmail.com; 3 Faculty of Chemistry, Nicolaus Copernicus University in Toruń, Gagarina 7, 87-100 Toruń, Poland; iglinski@umk.pl; 4 GPCHEM, Laboratory of Biogas Research and Analysis, Legionów 40a/3, 87-100 Toruń, Poland

**Keywords:** bioplastics, chemical recycling, pyrolysis, waste management, circular economy

## Abstract

Plastics-based materials have a high carbon footprint, and their disposal is a considerable problem for the environment. Biodegradable bioplastics represent an alternative on which most countries have focused their attention to replace of conventional plastics in various sectors, among which food packaging is the most significant one. The evaluation of the optimal end-of-life process for bioplastic waste is of great importance for their sustainable use. In this review, the advantages and limits of different waste management routes—biodegradation, mechanical recycling and thermal degradation processes—are presented for the most common categories of biopolymers on the market, including starch-based bioplastics, PLA and PBAT. The analysis outlines that starch-based bioplastics, unless blended with other biopolymers, exhibit good biodegradation rates and are suitable for disposal by composting, while PLA and PBAT are incompatible with this process and require alternative strategies. The thermal degradation process is very promising for chemical recycling, enabling building blocks and the recovery of valuable chemicals from bioplastic waste, according to the principles of a sustainable and circular economy. Nevertheless, only a few articles have focused on this recycling process, highlighting the need for research to fully exploit the potentiality of this waste management route.

## 1. Introduction

In recent years, the world has been involved in a transition process from the fossil–linear economy toward renewable–circular economy.

In this context, the reduction of the utilization of fossil-based plastics plays a significant role due to their carbon footprint, environmental pollution, and waste management problem. In fact, plastic materials have been involved in a process of exponential growth over the past few decades. From the 1950s of the last century, the excellent features and low costs of these oil-derived products have made them essential for a wide range of applications [1,2,3,4]. Nowadays, the production of traditional virgin plastic is more than 360 million tons yearly (55 in Europe) [5], and its manufacturing involves around 6% of the global oil production [6].

These data point out the impact of traditional plastics, which starts from the early stage of their life cycle due to their fossil-based feedstock and related emissions, and bioplastics are a great opportunity for reducing GHG emissions related to the use of traditional plastics [7].

The other main critical aspect of conventional plastic materials is their disposal. The enormous spread of this material has caused a huge amount of waste over the past few years. The durability of these items and the carelessness with respect to the end-of-life process have resulted in the “invasion” of plastic materials in the environment, creating great concerns about the “plastic pollution” phenomenon [7,8].

Bioplastics have been recognized as a possible solution to these issues [1,9,10,11,12,13]. According to “European Bioplastics” [14], the term “bioplastic” includes all plastic materials that have at least one of the following characteristics:

- It is made from biological feedstocks.
- It is biodegradable.

All the different bioplastics can be categorized into three different families:

1. Bio-based (or partly bio-based) plastics, non-biodegradable (bio-based PE, PP, PET,);
2. Bio-based and biodegradable plastics (PLA, starch blends, PHA, PBS);
3. Fossil-based biodegradable plastics (PBAT, PCL).

Bioplastics are usually considered more sustainable materials due to the advantages of not involving fossil sources (for families 1 and 2) in the production and/or the possibility of biodegrading them at the end of their life (for families 2 and 3) by reducing their environmental impact.

According to the European bioplastic forecast, the global bioplastic production capacity in the next 5 years will triple [14]. Moreover, production growth involves all the continents, in particular Asia (more than 300% increase) and Europe (more than 100% increase).

The EU promotes designs with easier recyclability, expanding and improving the sorting of different plastic waste to simplify logistics and ensure high quality for the recycling industry, while creating viable markets for recycled plastics [15]. The EU approach to bioplastics is also quite prudent [16], as bioplastics are considered “contributing to reduce ‘unavoidable’ littering, still not fully solving the littering problem of the single-use items”.

Another concern has been raised due to the lack of a clear framework on the actual biodegradability of these materials and the lack of awareness from consumers about the government guidelines for an efficient and correct way of recycling [17,18]. Several types of bioplastics are considered “biodegradable” (bioplastic family 2 and 3), and each of them has different biodegradability characteristics. The main problem is the distance between composting standards and the real operating conditions of industrial composting [19] or other common disposals for organic waste, such as composting at home, soil burial disposal, etc. [20,21,22].

This overview shows how the search for potential solutions to limit the environmental impact is complex and cannot be solved only by replacing plastics with bioplastics. According to the European strategy, material design is key to ensuring a valuable end-of-life pathway to minimize the utilization of virgin raw materials and resources and ensure efficient and effective disposal. Following the waste hierarchy [3], disposal is the final solution for ending the useful life of a product. Re-using, recycling and recovery must be preferred and need to be evaluated to better apply circular economy concepts.

When it comes to bioplastic materials, there are several types of recycling routes. Mechanical recycling—primary or secondary—is the “shorter” route for waste reuse. Primary recycling methods ensure the obtainment of products with the same characteristics of virgin materials; for bioplastics, they usually involve only manufacturing waste, as the use of waste materials would not provide the same performance as virgin feedstock [7,23,24]. Secondary recycling processes usually involve reprocessing and downgrading of bioplastic characteristics [7,23,25]. General reprocessing techniques include, for example, screw extrusion, injection molding, blow molding, etc. [23]. Chemical recycling techniques are often referred to as tertiary and consist of conversion of the bioplastic waste into chemicals that could be used as polymer precursors and/or chemicals for other purposes [7,23,26]. Different processes may be considered: pyrolysis or gasification separates chemical compounds by a thermal depolymerization of bioplastics, while solvolysis methods (hydrolysis, alcoholysis) operate by chemical depolymerization of the material [7,27]. Finally, energy recovery by incineration represents the quaternary recycling path [7].

In the scientific literature, many articles have been published on the degradation of biodegradable bioplastics, considering different materials, blends, biodegradation processes, and environmental conditions [19,28,29,30,31,32]. Bioplastic recycling has been analyzed in different review studies, but in general has focused either on a wide range of possible routes, providing an overview of the potential processes for all types of bioplastics [23,25,33,34], or on the potential recycling strategies for a single biopolymer [22,35,36,37].

Several LCA studies showed that mechanical and chemical recycling present considerable advantages in terms of the impact of global warming, environmental benefits and socioeconomic aspects compared to aerobic composting [22,38]. More in detail, most articles and reviews on LCA consider mechanical recycling as the favorite route in terms of environmental footprint [39,40,41].

Based on the above-mentioned considerations, this review contributes to the analysis of the state of the art and to clarify the potential perspectives of biodegradable bioplastics’ recycling processes with particular focus on thermal depolymerization, evaluating its role in circular economy practices. The focus is on biodegradable families. Among the different possible bioplastics, the four most widespread in the coming years have been considered, according to data provided by European Bioplastics: poly butylene adipate terephthalate (PBAT), poly(lactic acid) (PLA), starch blends, and polyhydroxyalkanoates (PHAs) [14]. Moreover, the availability of works in the literature that investigated alternative recycling routes for specific biopolymers was crucial for the collection of data and development of the review. For some of the most studied biopolymers with a large number of promising applications, such as poly(ε-caprolactone) (PCL), it was not possible to dedicate enough space due to the lack of work assessing the effectiveness of mechanical/chemical recycling processes [42,43].

A clear path starting from the physical–chemical properties of the selected bioplastics to their waste management performance is depicted to provide an overview of currently studied recycling routes with particular focus on the thermal depolymerization recycling processes, highlighting opportunities and advantages with respect to the biodegradation pathways, as well as gaps, and future needs for further development.

This article starts with a brief literature review of bioplastics (Section 2). Then, a general description of the considered bioplastics is given, providing a description of their characteristics, typical commercial blends, applications, and end-of-life options, such as biodegradation and mechanical recycling, to provide adequate information about these polymers and show the limits of the biodegradable end-of-life routes (Section 3). Section 4 describes the state of the art of thermochemical recycling processes of the analyzed polymers. Finally, all the information presented is summarized in the conclusions section, where suggestions for future developments are also proposed.

## 2. Methodology

**Figure 1:**
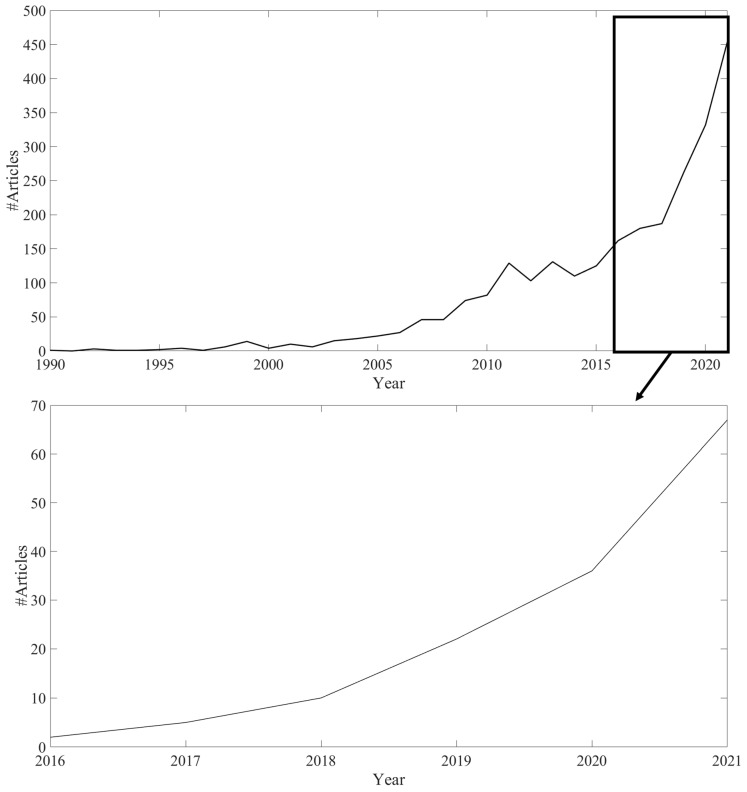
Annual number of articles on bioplastics. Source: Scopus.

**Figure 2:**
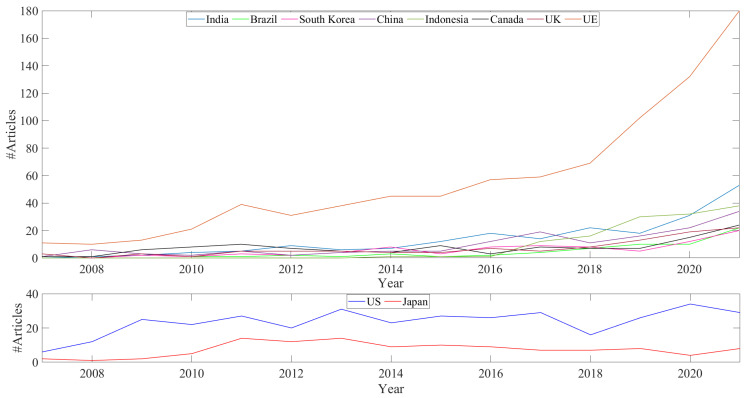
Annual contribution of articles on bioplastic field by most active countries. Source: Scopus.

**Figure 3:**
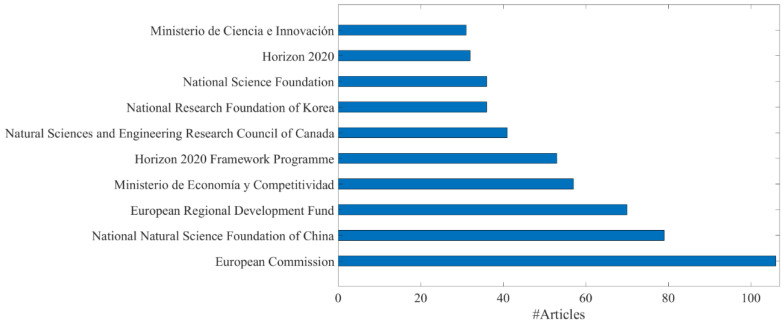
The top 10 funding sponsors of articles about bioplastics. Source: Scopus.

Bibliometric analysis of the Scopus database provides a clear trend in the bioplastic scientific literature. The number of articles with the term “bioplastic” in the title, abstract or keywords has increased sharply in the last 15–20 years (Figure 1). The acceleration in this field of study is not homogeneous around the world. In fact, as Figure 2 shows, the spread of articles is particularly evident in EU countries. Among these, Italy, Spain, and Germany emerged as more active in recent years. This strong interest is reasonably due to the political interest of the EU and of single national governments on the issue. Observing the top 10 funding sponsors of articles about bioplastics, six of them are EU countries or EU Institutions (Figure 3). Other countries with a growing scientific research interest in bioplastics are Canada, India, United Kingdom, Indonesia, China, South Korea and Brazil, while the United States and Japan seem to have a stationary trend after a peak of articles in the 2010s.

**Figure 4:**
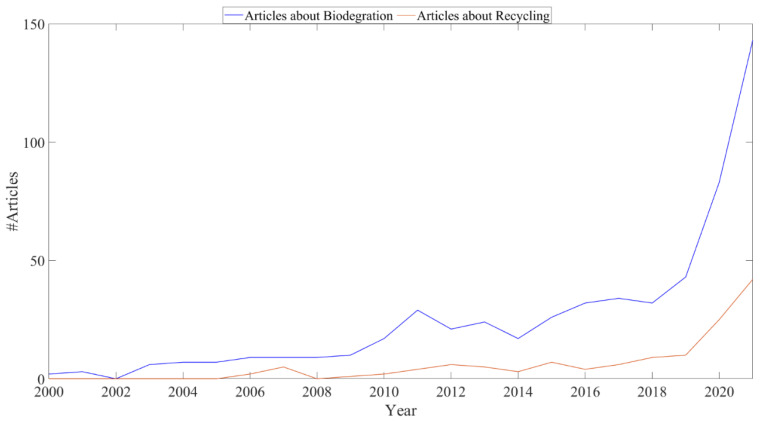
Annual contribution of articles in the bioplastics with the keywords: “Biodegradation” and “Recycling”. Source: Scopus.

It is also worth noting that the concept of ‘circular economy’ applied to bioplastics is relatively new, as it has been referred to more since the late 2010s, in particular in Italian and Spanish articles, which account for approximately 40% of the total number of articles (Figure 2). Then, it is also evident that the biodegradation process has been studied more than other recycling processes (Figure 4), highlighting the lack of a robust and detailed scientific framework around this topic.

## 3. Overview of the Most Widespread Bioplastics

In the following paragraphs, a general overview of the four more diffused biodegradable bioplastics is provided: PBAT, starch-based polymers, PLA and PHA. The main characteristics, uses and limits of the end-of-life management of these polymers are analyzed and reported.

### 3.1. PBAT

#### 3.1.1. PBAT Introduction and Characteristics

Among all fossil-based biodegradable plastics, one of the most widely used is poly (Butylene Adipate-co-Terephthalate), which is usually called PBAT. According to the data from European Bioplastics [14], in 2021 the global production capacities of PBAT have represented 19.2% of the overall bioplastic production (including: biobased nonbiodegradable, biobased biodegradable and fossil-based biodegradable), with more than 460 thousand tons per year produced [14].

It is a biodegradable synthetic aliphatic aromatic copolyester composted with 1,4-butanediol with both adipic and terephthalic acids [34,44,45,46,47,48,49]. The biodegradability of PBAT depends on the presence of the butylene adipate group [34,44,50] by increasing the susceptibility to hydrolysis and biological degradability [34]. The concentration of terephthalic acid is a trade-off between mechanical properties [34,46,47], which depends on the presence of aromatic acid in the copolyester and the reduction in its biodegradability, also caused by terephthalic acid. A balanced condition is obtained for the concentration of terephthalic acid below 40 wt.% [46,47] or 30–50 mol% [47].

Another crucial aspect for the biodegradability of PBAT is its amorphous structure characterized by a low crystallization [45]: biodegradability improves as crystallinity decreases [34]. Furthermore, due to its low crystallization, PBAT has low modulus and stiffness [34]; it is more flexible and has a greater elongation at break, with good processability properties, than other biodegradable polyesters [48,49,51,52,53].

PBAT has mechanical and thermal properties that change in a wide range, depending on the composition of the copolyester and the process of formation. In Table 1, some data about the polymer characteristics collected by different articles are reported, showing a significant variability of its characteristics.

Generally, these characteristics make it a good biodegradable alternative to low-density polyethylene (LDPE), suitable for a wide range of applications in plastic films [44,47,53,54,58,59,60]. Unfortunately, it has limits such as high costs [61], values three times the value of conventional polymers such as polyethylene and polypropylene [51], and low barrier capabilities to water vapor, oxygen, and carbon dioxide [58].

These aspects are the main drawbacks to the diffusion of PBAT. Currently, their effects are limited by the production of blends, ensuring lower costs and good mechanical properties while maintaining the matrix biodegradability.

#### 3.1.2. PBAT Blends

Both industry and scientific communities have been exploring research on various aspects of PBAT blends. Several companies in different countries have developed PBAT-based material, such as, for example: Mater-Bi by Novamont in Italy, Ecoflex by BASF in Germany, Biomax by DuPount in USA, Biotech by Biotech in Germany, and others [50,61,62]. In the recent scientific literature, many types of blends have been studied to evaluate new ways to improve the widespread diffusion of PBAT applications.

Lule et al. in [51] studied the PBAT blend with various concentrations of coffee husks, noting an increase in hydrophobic behavior and better mechanical properties of the compound and a decrease in polymer cost up to 32% for 40 wt% of coffee husks in the matrix [51]. Li et al. in [63] focused on the unsatisfactory water vapor barrier properties of PBAT films, preparing nanocomposite films containing organically modified montmorillonite (OMMT), via film blowing or biaxial orientation.

Some studies focused on the possibility of integrating PBAT and poly(lactic acid) (PLA) (PLA/PBAT blends) to investigate the compatibility of the blend. Li et al. in [48] prepared films of PLA/PBAT blend using a small amount of chain extender containing epoxy functional groups (ADR 4370F) to enhance compatibility of the matrix. In the study, an improvement in mechanical properties was found such as elongation at break, tensile strength, and tear strength, suggesting suitability for applications in shopping bags [48]. Mallegni et al. in [57] prepared blown films from the PLA / PBAT blend using as plasticizer and compatibilizer polypropylene glycol diglycidyl ether (EJ400) and nucleating agent (LAK 301) to allow good control of the extrusion process. The best tearing performance was obtained in the blend PLA/PBAT mix with 2% LAK. The tearing resistance obtained is higher than that of polypropylene, but still much lower than that of LDPE [57]. Schneider et al. prepared blown films of PLA/PBAT blend where the PLA was modified as epoxy-functionalized poly(lactide) (EF-PLA) due to the high reactivity of epoxy groups with the PBAT. The main results consisted of an increase in the maximum amount of PLA in the matrix (up to 70% wt. for 10 wt.%) and a general improvement in mechanical properties for 40% wt. PLA such as dart resistance (up to 400%) [57].

#### 3.1.3. PBAT Applications

Due to its mechanical characteristics, PBAT and its blends have been used for plastic film applications such as food packaging, trash bags, film wrapping, diaper back sheets, cotton swabs and mulch film [19,29,44,46,53,59,61]. However, the main problem is the offset between the standard certification criteria and the real conditions of organic waste management. This offset drives problems in end-of-life management of bioplastics. In fact, the requirement EN 13432 for biodegradability and composability is far from the mean condition in European industrial composting plants operating under thermophilic conditions (58 ± 2 °C) for 20 days, followed by a maturation phase (37 ± 2 °C) of approximately 40 days [19], or from the operating conditions achieved, for example, in soil-buried degradation. Then, several studies have been focused on characterizing the biodegradation properties of PBAT and blends to look for potential improvement of the current state of the art of the end-of-life management of this polymer [19,21,29,64,65]. An overview of these studies is reported in the next section.

#### 3.1.4. PBAT Biodegradation

One of the main routes for biodegradable polymers is composting. Regarding that, Ruggero et al. [29] monitored Mater-Bi® degradation under different composting conditions (20% starch, 10% additives, and 70% PBAT), finding that PBAT was the component more sensitive to moisture and temperature.

The most affecting parameter for PBAT biodegradation was the moisture content. In a report, [29] authors showed that moisture must be higher than 40% during the period of degradation (thermophilic and maturation phase), and below this level the biological activity showed a progressive slowdown until moisture content was approximately 25%, which represents the limit for the final stop of the process [29]. The authors also noticed that PBAT in Mater-Bi^®^ was subject to a higher degradation than that of pure PBAT. They justify their observation by the generation of cavities in to matrix due the faster degradation of starch [19]. Another aspect highlighted in [19] was the discrepancy between the standard conditions (e.g., EN 13432) and the industrial composting conditions. In their study, they analyzed the mean conditions in Europe and conducted an experiment at lab scale. Results showed that for a proper bioplastic, disposal management is necessary to guarantee time longer than the time required for the composting of the other organic waste.

Relatively to the anaerobic digestion process, Wei Peng et al. [64] observed that the addition of PBAT in a food waste matrix does not provide any advantages in terms of enhancing biogas production, and the material degradation rial occurred only under thermophilic conditions. This has been confirmed by Octavio García-Depraect et al. who showed in their work [21] how PBAT does not degrade significantly in anaerobic mesophilic conditions.

Finally, another possible route for the biological degradation of PBAT is enzymatic degradation. Kanwal et al. in [65] analyzed the decomposition of PBAT via enzymatic degradation. They realized rectangle-shaped pieces of about 20 mm length, 10 mm width and 0.7 mm thickness of PBAT samples. They are immersed in a separate tube containing 12 mL of phosphate-buffered saline with lipase B from Candida antarctica (6 mg mL^−1^) and incubated at a constant-temperature oscillator at 45 °C. The result showed that after 12 days the mass loss rate of the sample reaches 15.7%, significantly higher with respect to the black sample. The effectiveness of enzymatic degradation is confirmed by several analyses, such as the reduction of the temperature of thermal stability by a thermal gravimetric analysis, the weakening of the peaks registered by the FITR, and, finally, the X-ray diffraction confirms the decreases in the amorphous phase of PBAT.

#### 3.1.5. PBAT Mechanical Recycling

Only a few studies explored the potential [66,67] of different approaches for waste management of PBAT. La Mantia et al. [66] studied the mechanical recycling of a PLA/PBAT blend using a single screw extruder. The work highlighted how the predrying of the sample enhanced the potential of recycling because of the absence of hydrolysis degradation. The study concluded that five extrusion steps do not significantly decrease the mechanical properties of the blend, enhancing the possibility of using this solution as the end-of-life treatment of PBAT [66].

Oliveira T. et al. in [67] analyzed the mechanical recycling of a blend of biobased and biodegradable polymers (PBAT and thermoplastic starch) and fossil based non-biodegradable plastic, polypropylene (PP), in order to prove the increase in mechanical recyclability with respect to pure material. During experiments carried out in a single-screw extruder, the PP/PBAT–thermoplastic starch blend also shows good characteristics after seven reprocessing cycles, even better than the pure PP characteristics.

### 3.2. Starch Based Biopolymers

#### 3.2.1. Overview and Characteristics

Starch is one of the first biopolymers used for the development of sustainable materials to replace petroleum-based synthetic plastic production. Due to their low cost, renewability, and inherent biodegradability, starch-based polymers are high-potential feedstocks for the large-scale production of bio-plastic films [68,69]. However, poor physical properties, such as brittle structure, low mechanical strength, high gas permeability and reduced water barrier resistance, are shortcomings that require physicochemical modification of the native starch structure as addition of plasticizers, inclusion of fibers/nano-particles or blending with other polymers [69,70,71].

Starch granules consist almost entirely of two main polysaccharides, namely amylopectin, accounting for 70 to 85% of total starch and amylose, present for the remaining 15 to 30%. The relative abundance of amylopectin and amylose can differ significantly between various starch sources [72,73]. Starch granules consist of a semi-crystalline structure with a central amorphous region, mainly composed of amylose, and a circumferential repetition of alternating crystalline and amorphous lamellae [74,75].

Due to its high brittleness and poor mechanical properties, native starch cannot be directly processed as thermoplastic material [76,77]. Plasticizers are generally used to increase the capability of processing starch-based biopolymers. Water is the most used plasticizer for starch, but other substances such as polyols (glycerol, glycol, sorbitol), nitrogen-containing compounds (urea, ammonium derived, and amines), and citric acid have found intensive use [77]. Thermoplastic starch (TPS) results in a flexible and processable material, recognized as one of the most promising materials for the large-scale production of biodegradable materials [72,77,78,79].

Chemical modifications are advantageous methods to further increase the functionality of the modified starch. Oxidation, esterification and etherification are the main chemical modification methods, all based on the reaction of free hydroxyl groups of glucose monomers with a functional group, such as organic chloro-compounds, acid anhydrides, epoxy and ethylenic compounds [72,80,81]. These modifications lead to an improvement of native starch properties such as solubility in water, swelling, and retrogradation characteristics. Chemically modified starches with stabilized properties have a potential application on a large scale in the drug delivery system, pharmaceutical and food industry [81,82].

#### 3.2.2. TPS Blends

Although TPS has better characteristics with respect to native starch, it is generally unable to meet market requirements [70,83,84].

The most widely reported reinforcing methods are the incorporation of fibers/particles into the starch-based matrix and the blending of TPS with other renewable/fossil-based plastics or biopolymers; however, this work includes only the combination of intrinsic biodegradable polymers [70,79,85,86]. Although fiber/particle inclusions will be presented as distinct methods, some studies use combinations of these two strategies to obtain the best performing biomaterials [87].

Polymer blending is a simple method to tailor TPS properties to their intended end use [83,84]. Commercial films are obtained from the blend of TPS with other thermoplastics as poly(vinyl alcohol) (PVA or PVOH) or biodegradable hydrophobic polyester such as poly(lactic acid) (PLA), polybutylene succinate (PBS), poly(butylene adipate-co-terephthalate) (PBAT), polycaprolactone (PCL) and poly(3-hydroxybutyrate-co-3-hydroxyvalerate) (PHBV) [84,88].

#### 3.2.3. TPS Applications

Food packaging is the sector in which starch-based bioplastics have a more extensive application. Packaging requires different properties depending on the specific function that the film has to perform: rigid packaging needs high mechanical strength and toughness, long-life food requires high water/oxygen barrier properties, while films in contact with fresh products such as fruits, vegetables, and meat demand high permeability, characteristics that can be guaranteed by thermoplastic starch films [72,84]. The high hydrophilicity of TPS films is a property that limits their extensive use for fossil-based plastic replacement [71]. Chemical modifications are particularly effective in improving the barrier properties of TPS films. Several studies report how the biocompatibility and biodegradability of starch-based bioplastics are important properties that demonstrate the potential application of TPS films in the delivery of drugs, pharmaceuticals, and antimicrobial materials [89,90].

#### 3.2.4. TPS Biodegradation

Table 2 resumes the main findings about mechanical, barrier properties, and biodegradability of starch-based films. All the studies show how the various techniques to modify the native starch structure, such as starch plasticization, chemical modification, inclusion of natural fibers reinforcement or blending with other polymers, are effective in improving mechanical and barrier properties.

Abera et al. investigated the effects of different types and their concentration on anchote starch films realized [91]. Plasticized films with 1-ethyl-3- methylimidazolium acetate showed higher flexibility, while the sorbitol film resulted in the highest tensile strength and modulus of elasticity. Ilyas et al., Jumadin et al., Li et Al, Oluwasina et al. and L. Ten et al. analyzed the effect of the inclusion of fillers, fibers, and nanocrystals of natural or modified biopolymers into thermoplastic starch matrices [68,69,85,92,93]. For all of these TPS composites, the inclusion of a reinforcing element had a positive effect on mechanical properties, more than doubling the tensile strength value, except for Li et al. and Oluwasina et al., where the reinforcing effect of the nanocrystals of maize starch and oxidized cassava starch on the thermoplastic pea and cassava starch, respectively, had a lower effect.

Soil burial tests outlined a general biodegradability of all the samples. Ilyas et al. and Oluwasina et al. found similar degradation rates for thermoplastic starch films without reinforcing elements, but the addition of fillers had a different impact on biodegradability: in Oluwasina et al., the biodegradation rates of oxidized cassava starch experienced a significant reduction [68], while in Ilyas et al., nanofillers of sugar palm nanocrystalline cellulose had a smaller impact on the sugar palm–starch matrix biodegradation [69]. Jumadin et al. showed slower biodegradation rates in comparison to the other studies of this class of natural fillers/fibers-reinforced materials.

Priya et al. and Kenny et al. studied the reinforcing effect of PVA on pea and corn starch matrix, respectively [94,95]. Both studies found out how mechanical properties of PVA–starch blends, obtained with a casting solution method, are affected by the variation of PVA/TPS ratio and type of plasticizer. A soil burial test was performed by Priya et al., results highlight a weight loss of the samples of 45.65% in 120 days, showing how various modifications have a consistent effect on the original starch biodegradability [94].

Del Rosario Salazar-Sánchez et al., Palai et al., Sanyang et al., Lv et al. and Ocelić et al. realized and tested thermoplastic starch blends with PLA [83,96,97,98,99]. All the studies include significant amounts of PLA in the blends, ranging from 22% to 80%.

PLA inclusion in the polymeric composite had a positive effect on the mechanical properties, but there are serious drawbacks in terms of biodegradability of the films. Del Rosario Salazar-Sánchez et al. studied the structural change in 22/78 (% wt) PLA/TPS composite during biodegradation and found a significant mass loss, 65% wt in 32 days, a biodegradation rate that is not significantly influenced by the presence of PLA and is able to match the standard required for aerobic composting processes.

Palai et al. highlighted limited biodegradability of their TPS/PLA blend, after having performed a soil burial test for three months. They reported a 40.06% of mass loss after 90 days, a biodegradation rate considered high with respect to the common rate of PLA, obtained due to the increase in contact area of water and microorganisms for the PLA component due to the early biodegradation of starch [97]. Lv et al. found a very limited biodegradability of TPS/PLA blends reinforced with wood flavor fillers, with weight loss that varies for the samples analyzed, according to the various structures and properties, but is limited, showing a very partial biodegradation after 105 days [99]. Magalhães et al. realized thermoplastic corn starch blends with PHBV, reinforced with organically- modified montmorillonite as compatibilizer [100]. Inclusion of cloiside 30B (30B) resulted in an important improvement of the mechanical properties of the film, due to the increase in interfacial adhesion between PHBV and starch, and to the reduction in particle size [100].

**Table 2:** Mechanical, barrier properties and biodegradability of TPS-based biopolymers.

Biodegradation	Water Solubility (%)	Water Vapor Permeability 10^−10^ *g*/(*s·m·Pa*)	Elongation at Break (%)	Elastic Modulus (Mpa)	Tensile Strength (MPa)	Process	Plasticizer/Additives	Reinforcement	Starch Source	Ref.
85.76% (wt loss) after 9 days	33.36	9.58	38.1	53.97	4.8	Solution Casting	Glycerol/Sorbitol		Sugar Palm	[69]
74.8% (wt loss) after 9 days	18.45	8.17	24.42	178.83	11.47	Solution Casting	Glycerol/Sorbitol	0.5% (wt) Nanofillers of Sugar Palm Nanocrystalline Cellulose	Sugar Palm	[69]
	20.97		48.95	133	6.35	Solution Casting	Glycerol		Anchote	[91]
	31.34		25.43	1200	15.3	Solution Casting	Sorbitol		Anchote	[91]
29.95% (wt loss) after 4 weeks				128.72	1.89	Compression Molding	Glycerol		Cassava	[85]
26.22% (wt loss) after 4 weeks				285.3	5.05	Compression Molding	Glycerol	5% (wt) Cogon Glass Fibers	Cassava	[85]
			22.34	95.93	4.28	Melt Extrusion	Glycerol		Wheat	[83]
			2.14	1119.21	24.26	Melt Extrusion	Glycerol	50% (wt) PLA	Wheat	[83]
			432.52	287.79	10.5	Melt Extrusion	Glycerol	50% (wt) PCL	Wheat	[83]
Complete biodegradation after 60 days			0.78	55.88	0.46	Manual Molding	Glycerol	Sugarcane Bagasse	Cassava	[101]
Complete biodegradation after 60 days			0.74	74.32	0.57	Manual Molding	Glycerol	Sugarcane Bagasse/Cornhusk (14/6)	Cassava	[101]
Complete biodegradation after 60 days			0.44	52.6	0.37	Manual Molding	Glycerol	Sugarcane Bagasse/Malt Bagasse (16/4)	Cassava	[101]
Complete biodegradation after 60 days			0.63	43.7	0.33	Manual Molding	Glycerol	Sugarcane Bagasse/Orange Bagasse (16/4)	Cassava	[101]
	22.35	7.92	56.81	38.38	3.12	Solution Casting	Glycerol		Corn	[93]
	26.23	1.85	125.22	10.65	6.43	Solution Casting	Glycerol	61% (wt) Chitosan	Corn	[93]
		11.18	29.23	21.15	5.76	Solution Casting	Glycerol		Pea	[92]
		4.26	12.58	85.72	9.96	Solution Casting	Glycerol	5% (wt) Maize Starch Nanocrystals	Pea	[92]
		5.3	11	420	14.2	Solution Casting	Glycerol		Pea	[95]
		3.5	160	210	14	Solution Casting	Glycerol	PVA/Pea Starch (2/1)	Pea	[95]
			149		14.94	Solution Casting	Citric Acid	PVA (PVA/Corn Starch 1:1)	Corn	[94]
45.65% (wt loss) after 120 days			182.27		38.56	Solution Casting	Citric Acid/Glutaraldehyde (Cross-linker)	PVA (PVA/Corn Starch 1:1) and 20% (wt) Grewia Optiva Fiber	Corn	[94]
65% (wt loss) after 32 days						Extrusion Blow-Molding	Glycerol/Anhydrous Malic Acid (Compatibilizer)	PLA 22%(wt)	Cassava	[96]
	32.75	6.37	46.66	169	7.74	Solution Casting	Glycerol/Sorbitol (1/1)		Sugar Palm	[76]
	23.91	0.33	21.02	312	12.07	Solution Casting	Glycerol/Sorbitol (1/1)	PLA 40%(wt)	Sugar Palm	[76]
	19.28	0.21	15.53	324	13.65	Solution Casting	Glycerol/Sorbitol (1/1)	PLA 50%(wt)	Sugar Palm	[76]
40.06% (wt loss) after 90 days			6.4	1021	23.5	Extrusion Blow-Molding	Glycerol/GMA(Grafting agent)/BPO (Initiator)	PLA 80%(wt)	Cassava	[97]
6.15% (wt loss) after 105 days					46.41	Injection Molding		PLA 70%(wt) and Wood Flour Fillers 21% (wt)	Corn	[99]
11.23% (wt loss) after 105 days					44.63	Injection Molding		PLA 70%(wt) and Wood Flour Fillers 9% (wt)	Corn	[99]
			7	95	12	Extrusion	Glycerol		Potato	[88]
			185	12	10.2	Extrusion	Glycerol	PBAT 40% (wt)	Potato	[88]
			80	58	12.3	Extrusion	Glycerol/PBATg and MA (Compatibilizer 2% wt)	PBAT 40% (wt)	Potato	[88]
			3.21	375.5	6.89	Extrusion Compression-Molding	Glycerol	PHBV 50% (wt)	Corn	[100]
			2.23	827.3	12.64	Extrusion Compression-Molding	Glycerol	PHBV 50% (wt)/C30B	Corn	[100]

#### 3.2.5. TPS Mechanical Recycling

In some of the studies reported in Table 1, soil burial tests were performed to assess the biodegradability of thermoplastic starch polymer composites; the studies concluded that the greater the modification to the native structure are, the greater the impact on the biodegradability of the samples tested.

In the literature, some studies have been presented on the mechanical recycling of thermoplastic starch biopolymers. Ibáñez-García et al. demonstrated that commercial Mater-Bi starch-based biopolymer can be reprocessed four times by injection molding without the addition of virgin material [102]. In the study, reprocessing did not have a significant effect on the strength of the composite, but a negative impact on toughness [102]. Moreover, the authors realized a Mater-Bi composite filled with 20 wt% almond shell powder (ASP) and epoxidized linseed oil (ELO) as a compatibilizer additive and tested the mechanical recyclability of the film. Test results outlined that TPS/ASP composite could be recycled up to six times but with a more critical impact on mechanical processes. Lopez et al. found that thermoplastic starch cannot be recycled with injection molding processes more than twice due to serious degradation of ductility and a complete loss of plasticity [103].

### 3.3. PLA Introduction and Characteristics

#### 3.3.1. Overview and Characteristics

Polylactic acid or polylactide (PLA) is a biobased, biodegradable, widely used bioplastic. It is a linear thermoplastic aliphatic polyester synthesized from lactic acid molecules [7,25,35,104,105,106].

In 2021, global production capacities of PLA covered 19.2% of overall bioplastics [14].

Lactic acid is obtained by fermenting sugar contained in various sources, such as: corn starch, sugar beet, tapioca roots, potato starch, and others. [7,25,35,104,105].

Lactic acid is a chiral molecule that can exist in three different stereochemical forms: L-lactide (PLLA), D-lactide (PDLA) and D-L-lactide (or meso lactide) (PDLLA) [7,25,104,106,107]. The ratio utilized for these isomers determines the overall properties of future synthetized PLA [25]; generally, commercial PLLA has a small amount of D-lactide (2–4%) [7].

PLA is a thermoplastic, water-insoluble, high-strength, and high-modulus polymer. Its peculiarity is that the adjustment of the composition of lactic acid monomers allows for control of the molecular weight and the crystalline structure [7,108]. Higher molecular weight drives higher glass transition and melting temperature, as well as greater tensile strength and elastic modulus [23].

The main limits of PLA are its brittleness, low resistance to heat, and slow crystallization rate [23,105,107]. However, PLA, as the most biodegradable thermoplastic polymer, has features comparable to petrol-based plastic such as polystyrene (PS) and polyethylene terephthalate (PET), polyvinyl chloride (PVC), low-density polyethylene (LDPE) [105,106] which can be replaced in different uses.

In Table 3, some data about the polymer characteristics collected from different articles are reported, showing a significant variability of its characteristics.

#### 3.3.2. PLA Blends

Statistics show a growing interest in PLA-based blends, in particular, blends with other biodegradable polymers [114]. One of the most frequently investigated problems is related to PLA blends that increase the degradability of the material. For this purpose, starch is a good biopolymer. In fact, as previously described, it has a greater biodegradability and it is cheaper than PLA [106].

T. Ke et al. studied the interaction between PLA and starch; they observed that the water absorption of the blends increased with starch addition. Furthermore, the crystallization rate and the degree of crystallinity increased as well, while the melting temperature decreased [104]. The addition of poly(ethylene glycol) (PEG) as a plasticizer for PLA/starch blends was evaluated in different articles [115,116,117]. This enhanced PLA crystallization, improving ductility and toughness.

The combination with PBAT has also been investigated. Jiang et al. [118] studied the mechanical properties of a PLA/PBAT blend obtained by a twin-screw extruder. They showed how the addition of PBAT increased toughness and elongation at break but negatively affected tensile strength and modulus. Moreover, PBAT increased the crystallization rate. Some other studies [119,120] showed how PBAT in the mixture increased the ductility of the materials, up to 300% for a PBAT content of 25%.

#### 3.3.3. PLA Applications

PLA and its blends are usually employed for packaging applications, compost bags, and food and beverages such as disposable tableware, plates, cups, and bottles. Moreover, in the form of fibers and non-woven textiles, it also has several applications such as upholstery, disposable garments, and awnings [7,105,106]. Other particular fields of use are biomedical applications and 3D printing. In fact, due to its biocompatibility, it is widely used in the biomedical and healthcare fields for drug delivery microspheres, sutures, bone fixation materials, stents, tissue engineering, feminine hygiene products and nappies [25,106].

However, some drawbacks limit its widespread diffusion. The production cost is still higher than conventional or other biobased plastic (such as starch) [23,35,106]. The expensiveness of its production begins with the earliest fermentation of sugar. In fact, the result of this process is often insufficient purity and requires additional processes to obtain a lactic acid suitable for PLA production [23,35]. This first phase reaches up to 50% of the total production cost [35]. Furthermore, PLA has mechanical limits, such as its brittleness, limited service temperature range and limited gas barrier properties, which also limits its use [105,106,121]. However, the main problem with PLA is its poor biodegradable behavior, which makes it one of the hardest bioplastics to decompose.

#### 3.3.4. PLA Biodegradation

PLA degradation of PLA occurs through hydrolysis of the ester bond [34,108] and the biodegradation activity of aerobic and anaerobic microorganisms [25,122,123]. The main parameters that affect its degradation are: temperature, humidity, size, and shape of the samples. At ambient temperature, biodegradation is slow and requires up to 2 years for complete degradation [23]. This makes the polymer unsuitable for soil or domestic composting [25,35]. The high humidity environment promotes the hydrolysis and growth of biodegradation microorganisms [34,122,123]. The ideal condition for PLA degradation requires thermophilic conditions at which degradation is achieved between 90 and 120 days [23,107,122]. Mainly, the PLA degradation is promoted by blending it with other bioplastics that are more easily degradable bioplastic and by mixing it with other organic compounds [34].

Wei Peng et al. [64], as already described for the PBAT bioplastic, found that the addition of PLA in a food waste matrix does not provide any benefits to improve biogas production. Additionally, for PLA, visual degradation of the material was observed only in thermophilic condition.

Bandini et al. [124] analyzed the degradation performance of a sample containing 30% PLA in bio-waste matrix after 25 days of hydraulic retention time under thermophilic conditions.

#### 3.3.5. PLA Mechanical Recycling

Due to its low degradation rates with respect to the other bioplastics, PLA has received more attention in the scientific literature by exploring alternative pathways for the end-of-life process. PLA soil biodegradation can take years with the risk of increasing environmental pollution [22,125,126,127]. An accurate control of the conditions of the composting process in terms of temperature and humidity is required for a correct biodegradation of PLA, feasible only in industrial applications, different from residential ones [35,128].

Mechanical recycling is a widely studied solution and represents an effective alternative to the biodegradation of PLA. Many authors have studied, using various analysis techniques, the impact of reprocessing on the structure and composition of PLA polymers and the consequent changes in mechanical, thermal, and optical properties [37].

Cosate de Andrade et al. studied the effect of reprocessing PLA waste on the thermal and mechanical properties in a single-screw extruder. The impact of a chain extender on the physical structure and related properties was also deepened [128]. The results show that extrusion has a low effect on the tensile strength of the samples and a remarkable influence on the Young modulus, leading to an increase in crystallinity of more than 22%, resulting in a stiffer polymer [128].

Yarahmadi et al. analyzed the impact on mechanical, thermal and rheological properties of multiple processing of PLA blends with non-biodegradable HDPE and PC using a modular twin-screw extruder [129]. An interesting outcome of the research is that the aging cycle had significant consequences on the recyclability of the polymer, and it was not possible to recycle the polymer over one cycle. However, multiple processing instances in PLA/HDPE and PLA/PC blends, not subjected to aging cycles, do not significantly affect the Young modulus of the materials, with a slight increase and decrease in elongation at break, respectively [129].

### 3.4. PHA

#### 3.4.1. PHA Overview and Characteristics

Polyhydroxyalkanoates (PHAs) are bio-based polyesters accumulated by more than 75 different species of bacteria as energy and carbon storage in the cell [1,2].

Potential production from abundantly available renewable resources, biodegradability in both the soil and marine environment, and the intrinsic biocompatibility make PHA biopolymers attractive to replace fossil-based plastics in a wide range of applications [130,131,132,133,134].

More than 100 different monomers have been recognized as the basis for PHA, allowing this type of biopolymer to have a wide range of properties [117].

PHA production consists of a fermentation step, where bacteria growth and polyester accumulation take place in a bioreactor under controlled conditions, and a recovery step, where various techniques, i.e., solvent extraction, floatation or digestion method, are employed for cell breakage and polyester extraction [130,135].

PHAs are classified into the short-chain length class, characterized by monomeric building blocks with 3–5 carbons such as poly (3-hydroxyvalerate) (PHV) and poly (3-hydroxybutyrate) (PHB), and medium-chain class (monomeric units of 6–14 carbons), such as poly (3-hydroxyoctanoate) (PHO) [132,136,137]. Generally, short-chain PHAs are brittle and rigid and lack the mechanical properties to meet the requirements for food and packaging applications due to elongation at break, while medium-chain bio-based polyesters are elastomeric, but have reduced mechanical strength [132,136]. Secondary recrystallization with ageing is the principal cause of the weakness and brittleness of PHB, together with the high glass transition temperature and a narrow gap between the melting temperature (180 °C) and the thermal decomposition temperature (210 °C) that make PHB processing difficult [138,139].

To improve the mechanical and technological properties and bring PHA to industrial use, various techniques are employed, such as biological, chemical, and physical [140].

#### 3.4.2. PHA Blends

Physical modifications of PHAs are aimed at improving the mechanical properties of PHA-based biopolymers and lowering the production cost, which is several times higher than petroleum-based plastics [141]. Blending with natural materials such as starch, fibers, and cellulose derivatives is the most widely diffuse physical modification technique for PHAs. Table 4 resumes the main findings about blending of PHA with natural biopolymers. As can be observed in Table 4, the use of plasticizers and compatibilizers/cross-linking agents is very diffused practice to increase the processing capacity and improve the interfacial bond between natural fibers and the matrix, respectively [138,142]. The effect of agricultural waste loading on the mechanical properties of biopolymers is shown in Table 4.

C.M. Chan et al. reported a decrease in tensile strength with an increase in wood flour load in a PHBV matrix [143]. In constrast, C.S. Wu et al. observed a slight increase in mechanical properties with the load of rice husk in a compression molded PHA (g-AA) film [144]. The authors justified this trend with the enhanced dispersion of RH in the PHA-g-AA matrix, creating branched or cross-linked macromolecules [144]. However, L. Joyyi et al. experienced first an increase and then a decrease in the flexural strength of compressed molded films of P(3HB-co-3HHx) reinforced with increasing loads of kenaf fibers [145].

**Table 4:** Mechanical properties and biodegradation of PHA-based biopolymers.

Biodegradation	Flexural Modulus (Mpa)	Flexural Strength (Mpa)	Elongation at Break (%)	Elastic Modulus (Mpa)	Tensile Strength (MPa)	Process	PHA Blends/Reinforcement	Ref.
27% (wt) Mass loss after 60 days	-	-	16	350	-	-	PHA (g-MA)	[142]
62.5% (wt) Mass loss after 60 days	-	-	24	420	-	Compression Molding	PHA (g-MA) with 20% (wt) agent-treated palm fibers	[142]
82% (wt) Mass loss after 60 days	-	-	22	400	-	Compression Molding	PHA (g-MA) with 40% (wt) agent-treated palm fibers	[142]
-	-	-	38	302	10.2	Extrusion	Mater Bi Z Grade-PHA (95.5/4.5% wt/wt)	[146]
17.8% Mass loss after 86 days	-	-	-	-	-	Compression Molding	PHB	[147]
22.5% Mass loss after 86 days	-	-	-	-	-	Compression Molding	PHB with 2.5% PP-g-MA (wt) and 3% clay wt)	[147]
25.9% Mass loss after 86 days	-	-	-	-	-	Compression Molding	PHB with 5% PP-g-MA (wt) and 3% clay wt)	[147]
-	530	16.8	-	-	-	Compression Molding	P(3HB-co-3HHx)	[145]
13% Mass loss after 6 weeks	1610	21.2	-	-	-	Compression Molding	P(3HB-co-3HHx) with 30% (wt) of kenaf fibers	[145]
-	1820	12.2	-	-	-	Compression Molding	P(3HB-co-3HHx) with 40% (wt) of kenaf fibers	[145]
2.7% Mass loss after 12 months	-	-	-	-	32	Extrusion	PHBV	[143]
6.4% Mass loss after 12 months	-	-	-	-	29	Extrusion	PHBV with 20% (wt) of wood flour	[143]
12.5% Mass loss after 12 months	-	-	-	-	22	Extrusion	PHBV with 50% (wt) of wood flour	[143]
36% (wt) Mass loss after 60 days	-	-	-	580	16	Compression Molding	PHA (g-AA)	[144]
77% (wt) Mass loss after 60 days	-	-	-	550	17	Compression Molding	PHA (g-AA) with 20% (wt) of rice husk	[144]
92% (wt) Mass loss after 60 days	-	-	540	-	17.5	Compression Molding	PHA (g-AA) with 40 % (wt) of rice husk	[144]
-	-	-	3.9	-	12.5	Compression Molding	PHB with 30% (wt) of amylose starch	[139]
-	-	-	2.8	-	7.3	Compression Molding	PHB with 30% (wt) of amylopectine starch	[139]

#### 3.4.3. PHA Applications

Many studies have focused on PHA blends for their suitability for a wide range of applications. Synchronically, the requirements for specific applications are crucial for the selection of the optimal carbon source for microorganisms and downstream processing [130]. Due to intrinsic biocompatibility and non-toxicity, PHAs are optimal biopolymers for tissue engineering for medical/pharmaceutical applications [130]. Extensive research has been carried out on PHA for the construction of biodegradable scaffolds and the replacement of heart valves of living tissue [132].

#### 3.4.4. PHA Biodegradation

Biodegradation is considered the preferred disposal strategy for PHA-based biopolymers since they can be degraded both in soil (under aerobic and anaerobic conditions) and in marine environments, without the release of toxic products [117,138]. Various studies have reported that PHA biodegradation is influenced by an elevated number of factors, such as microbial activity of the environment (different bacteria produce different PHA-depolymerases to degrade PHAs), moisture, temperature, degree of crystallinity, pH of the environment, and exposed surface area [117,130,138].

The PHA structure itself influences the degradation rate of biopolymers in soil: copolymers with higher exposed and porous surface area and low crystallinity have been found to degrade more rapidly with respect to homopolymers [148].

Table 4 reports the results of the soil burial tests for some biopolymers blended with natural materials. The burial test conditions and the equipment employed in the various studies are different, and hence the degradation rates reported for the PHA biopolymers are not in agreement. In various cases, after 60 days, PHB degradation exceeds 20% [142], while in other studies the mass loss of PHBV does not exceed 3% [147]. As outlined in other works, in the literature there is generally disagreement on the biodegradation rates for PHA-based biopolymers [149]. Otherwise, for all the studies reported, the inclusions of natural fillers/fibers have a positive impact on biodegradation, increasing the degradation rates in a few months.

#### 3.4.5. PHA Mechanical Recycling

With biodegradation in the soil as the designed cradle-to-grave route, there are limited studies in the literature for alternative PHA recycling routes [23].

Rivas et al. investigated the effect of multiple reprocessing cycles on PHB properties, assessing the extrusion process for mechanical recycling. PHB was heated up to 170 °C without the use of additives/plasticizers [150]. This study revealed that reprocessing had a strong effect on PHB mechanical properties, which degraded significantly after three cycles. These changes were attributed to the changes in the PHB structure, probably ascribed to the reduction of molecular weight due to chain scission reactions caused by thermal degradation [150].

## 4. Thermal Process for Biopolymers Recycling

The different biopolymers discussed above have peculiar behaviors with respect to biodegradation. Starch-based films show good biodegradation rates under controlled temperature and humidity conditions; however, the blending of starch with other polyesters has a high impact on slowing down the rate of biodegradation. For PBAT, PLA and PHA biomaterials, many studies report evident difficulties in biodegradation or limited biodegradation rates, opening up alternative recycling routes. Furthermore, biodegradation makes it hardly possible to recover energy and bioresources from PLA waste, in contrast to the main pillars of sustainable development and the circular economy [127,151].

Mechanical recycling is a widely studied end-of-life strategy for bioplastic materials, which leads to the conversion of waste into secondary raw material with limited impact on the original structure. However, the mechanical recycling process involves several steps before extrusion/injection, in which the feedstock must be separated and sorted, treated by washing and dried [37,152]. Bioplastic sorting is a crucial phase because even small traces of different biopolymers can have a significant impact on the efficiency of the entire process [25,27,35,153]. Moreover, highly efficient sorting technologies, such as near-infrared spectroscopy (NIR), with a separation efficiency of about 97.5%, require further improvement and are still not economically advantageous. Washing and drying are very expensive steps in terms of energy and water resources and represent an environmental risk of possible water contamination [154].

In chemical recycling processes, depolymerization reactions occur to degrade the polymer backbones into their monomers for a new synthesis and recovery of other highly valuable chemicals [23,37]. The principal advantage of chemical recycling over mechanical recycling is that it is tolerant of contamination of feedstock with other bio-plastics, and could potentially extend useful life for indefinite cycles [35].

Chemical recycling processes can be distinguished into processes where bioplastic matrices are depolymerized into their precursors immersed in organic solvents (often called solvolysis, such as hydrolysis and alcoholysis) and dry-thermal processes where biopolymers are depolymerized into their monomers and valuable chemicals in oxygen-free environments. For both solvolysis and thermal processes, feedstock characteristics and the operative condition deeply affect bioplastic conversion rates and product yield.

From an energy point of view, solvolysis processes are cost-effective because they require less energy input than thermal processes [23]. However, the flexibility of thermal processes with the variation of operating conditions and system design is an important aspect that makes it possible to drive the process toward the desired product (i.e., solid, liquid, or syngas). However, reducing the energy input of the process, recirculation, and combustion of syngas is an advantageous strategy [24].

**Figure 5:**
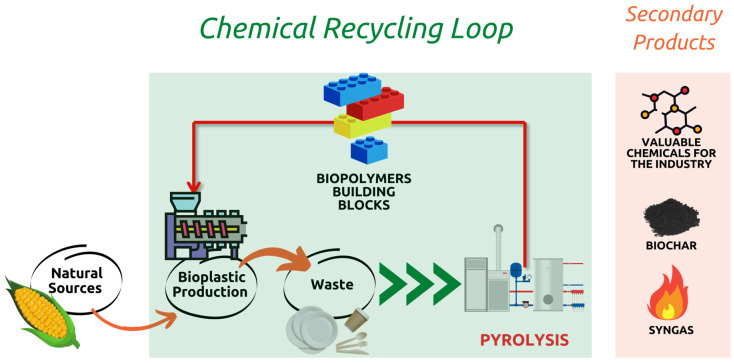
Concept of sustainable recycling of bioplastics waste by thermal process.

Moreover, the extensive use of solvents is an aspect that can have an environmental impact if not considered carefully [7]. Furthermore, compared to other chemical recycling technologies, the scale-up of pyrolysis to the industrial scale is more straightforward, as it is already considered a well-established technology with various commercial plants for biomass and plastic pyrolysis [24]. In combination with the production of building blocks for bioplastics, other products, such as valuable chemicals present in bio-oil, which are of great interest for the industry, biochar (solid fraction) and syngas, can be reused to sustain the energy requirements of the process or made available as a fuel for other use. Therefore, as reported in Figure 5, pyrolysis is described as a sustainable route, especially if the valorization includes most of the outputs of the process.

For all these reasons, over the last few years, several articles have proposed kinetic models for bioplastic heating depolymerization, leading to an estimation of the activation energy of the main reactions, providing possible strategies to make the process as cost-effective as possible [152,154]. Synergistic effects of biopolymers and biomass co-pyrolysis were also investigated, highlighting how the interaction between natural fibers/agro-industrial residues and bioplastics promotes the recoverability of precursors and valuable chemicals, reducing the activation energy of co-pyrolysis [155]. Only in some cases the studies included validation on laboratory scale reactors, which contributed to the assessment of the actual potentiality of the thermal process as an effective end-of-life route.

Undri et al. performed PLA pyrolysis tests in a microwave-assisted reactor, investigating the effect of microwave power, microwave absorber, and apparatus setup on yield and composition of the products [151]. The result shows a relevant presence of lactides in meso- and enantiopure-forms (more than 20% in most of the test conditions) both in solution and in crystal state. Moreover, they highlighted the positive synergistic effect of PLA with other polymers; tire microwave absorbers in this case led to a decrease in the yields of L-lactide crystals as a result of the high solubility of lactides into the resulting aromatic compounds contained in the liquid fraction that inhibited the formation of precipitate. The authors concluded that the co-pyrolysis of PLA with other plastics is not convenient if the objective is to recover L-lactide crystals, but it is still possible if chemicals or fuels identified in the pyrolytic oil are extracted and collected [151]. The results of this work are unique but require further deepening in the quantification of the exact amounts of chemical compounds identified in the pyrolytic oil and the understanding of the exact operating conditions to be applied on commercial-scale plants.

Saeaung et al. carried out PLA pyrolysis tests in a fixed bed reactor exploring the effect of the pyrolysis temperature in the range 400–600 °C and the catalyst effect of zeolite, spent FCC or MgO at a content of 20%, mixing the catalyst powder with PLA samples [156]. Only liquid and non-condensable gases were obtained at a temperature greater than 400 °C. The liquid and gas yields had a reverse trend with the temperature, probably as a result of secondary thermal cracking at high temperature, as suggested by the authors. The pyrolysis liquid phase recovered during the tested results in both wax and liquid form. An important result is the different selectivity of the various catalysts in relation to the major chemical species present in the liquid fraction: zeolite enhanced the lactide selectivity at 400 °C, increasing the relative area to 78.9%, while pyrolysis catalyzed by spent FCC did not result in lactide production, with a bio-oil rich in propionic acid. At 600 °C, instead, high yield L-lactic acid is obtained with a greater catalytic effect of spent FCC with respect to zeolite to drive the selectivity towards L-lactic acid at the expense of production of lactides [156]. The authors concluded that catalytic pyrolysis is an effective end-of-life route for biodegradable plastics to recover valuable chemicals.

**Figure 6:**
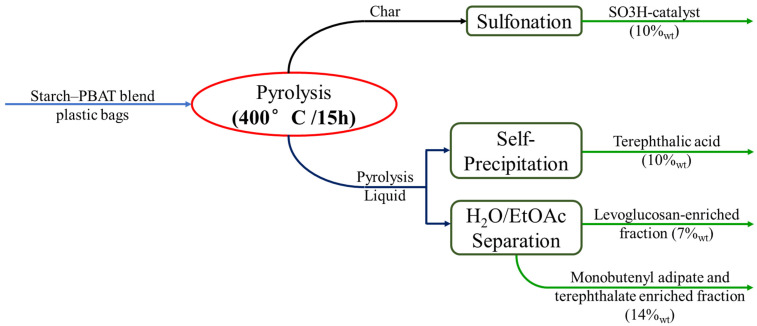
Sustainable approach for starch-based bioplastic waste valorization through slow pyrolysis process. Adapted from Samorì et al. [157].

Samorì et al. studied chemical recycling via slow pyrolysis of starch–PBAT blend plastic bags (70% of PBAT) for 15 h at 420 °C. The main products obtained by the process are summarized in Figure 6. The solid residue (yield 10% wt) was subjected to sulfonation to obtain a $SO_{3}H$-containing catalyst group heterogeneous catalyst (10 wt%) and was tested in the esterification of fatty acids with alcohols resulting in excellent reactivity. Highly pure terephthalic acid (4 wt%, 98.5% purity), an important building block in the chemical industry, self-precipitated in the liquid fraction. The remaining pyrolysis liquid was divided into two fractions: one water-soluble one, containing a relative abundance of levoglucosan of 46%, and other sugars/anhydrosugars, deriving from starch depolymerization, an ethyl acetate-soluble fraction enriched in monobutyl dicarboxylic acids [157]. The approach proposed by Samori is particularly effective in presenting a valid method for the separation of the different species identified in the oil, providing quantifications. The results are particularly attractive in terms of sustainable development of a production logic focused on recycling and saving fossil-based raw materials. However, further development and validation of the results on a larger scale is also required in this case, by carrying out a more detailed study of the optimal operating conditions of the process.

Various studies have proposed thermal degradation for the recycling of PHA waste into crotonic acid, a highly commercially distributed chemical that is currently produced by the petrochemical transformation of hydrocarbons into ethylene [158]. Moreover, crotonic acid can be re-polymerized into new PHA with both chemical and biological methods.

Ariffin et al. investigated the catalytic effect of magnesium oxide and magnesium hydroxide on the thermal degradation of PHB in a glass tube oven in the temperature range of 240–280 °C [159]. They obtained high condensate yield (over 80%) with a remarkable trans-crotonic acid yield, superior to 95% when a catalyst was used [159].

Similarly, Mamat et al. obtained a 50% trans-crotonic acid yield from pyrolysis of PHB inclusions using a glass tube oven setup [158]. They proposed a simplified model for the evaluation of the sale price for a bio-based production process of trans-crotonic acids based on fermentation and pyrolysis, demonstrating the economic feasibility of the proposed production route [158].

## 5. Conclusions

Optimization of end-of-life strategies for industrial and post-consumer waste of bioplastics has been the subject of debate and had particular attention in recent years. This review aimed to highlight the advantages and limits of different recycling routes for three of the most used bioplastics. In particular, stress has been placed on the link between the physical–chemical properties of the bioplastics and the most suitable conversion process. The study of the reported literature allows us to highlight the following main statements:

- It is evident that biodegradable bioplastics must be considered in their disposal, similarly to other materials. Each material needs an optimal end-of-life pathway to maximize the circular economy and the utilization of virgin raw materials.
- The cognitive bias that biodegradable bioplastics equates to a biodegradable end-of-life process needs to be overcome. Mechanical and thermal degradation recycling processes can significantly support the creation of best practices of the circular economy for these materials and must be evaluated to ensure optimal waste management strategies.
- Several LCA studies showed how mechanical and chemical recycling present considerable advantages in terms of global warming impact, environmental benefits, and socio-economic aspects with respect to aerobic composting. Among the various LCA studies found, several works focused on the comparison among various end-of-life pathways for PLA, concluding that high GHG savings can be attributed to mechanical or chemical recycling for the substitution of virgin PLA, underlining how the prevention of biomass cultivation to produce PLA precursors leads to environmental benefits.
- Further experimental data are required to evaluate more accurately the best recycling alternatives, in particular starch-based, PHA and PBAT bioplastics, considering the possible synergies between chemical and mechanical processes for optimized waste management routes.

As a result of the above-mentioned considerations, studies on the quantitative evaluation of the performance of chemical recycling routes for PLA, PHA, and PBAT bioplastics are crucial in finding optimal waste management processes for these materials, foreseeing the maximization of the effectiveness of the circular economy pillars. Pyrolysis, in particular, seems promising as a thermochemical route. Very few papers have been published on such a topic, with little to no attention on the relation between design, selectivity toward precursors, energy efficiency, and integration to other technologies for energy or production purposes. Higher research intensity on these aspects will lead to significant cost reduction, which is, in fact, heavily related to reactor design, calling for efficient continuous and easily scalable reactors. These, in turn, will require smart design solutions to guarantee stable operating conditions by varying feedstocks, while also considering the blending among bioplastics and other residual wastes.

## Figures and Tables

**Table 1:** Data collected for PBAT bioplastic.

T Melting (°C)	T Glass (°C)	Elongation at Break (%)	Elastic Modulus (Mpa)	Tensile Strength (Mpa)	Process	Plasticizer/Additivities	Blends	Ref.
		600	117.3	14.2			PBAT	[46]
115–125		670		21			PBAT	[44]
110–115	−30	>500	52	9			PBAT	[54]
130.4		330	52	15.5			PBAT	[51]
114	−34.1	927	38.9	11			PBAT	[55]
		1252	122.3	49.9	Molded with a twin extruder		PBAT	[48]
							PBAT	[56]
122.01		330	3950	47		Epoxy for PLA functionation	PBAT + PLA (60–40%)	[56]
149.1	−33	181	2100	13.7	Melt blended using a conical twin-screw extruder	Polypropylene Glycol di Glycidyl Ether (EJ400) 10% nucleating agent (LAK 301) 2%	PBAT + PLA (67−23%)	[57]
116	−26.3	312	94.2	8.3		Acetic Anhydride for modification of Cellulose Nanocrystal	PBAT + Cellulose Nanocrystal (98–2%)	[55]
129.7		500	118	17.2	Molded with a twin extruder	Coffee Husks surface-treated by a chemical silanization	PBAT + Coffee Husks (64–40%)	[51]
124.8	−31	730	349.2	36.4	Two-step reactive extrusion by a co-rotating twin-screw extruder	PBAT modified grafting 3 wt.-% Maleic Anhydride	PBAT + Talc (70–30%)	[46]
167	−26.8	290	792.5	35.4	Molded with a twin extruder	Epoxy functions Joncryl ADR-4370F (0.15%)	PBAT + PLA (40–60%)	[48]

**Table 3:** Data collected for PLA bioplastic.

T Melting (°C)	T Glass (°C)	Elongation at Break (%)	Elastic Modulus (Gpa)	Tensile Strength (Mpa)	Process	Molecular Weight g * mol^−1^	D-PLA%	Blends	Ref.
130–180	60–65	2–10	2.7–16	15.5–150				PLA	[109]
170–200	55–65	2.5–7	0.35–3.5	21–60		66,000		PLA	[110]
210	57	6	3.4				D-PLA (3–4%)	PLA	[111]
		5.4		40.8	Two-stage melt polycondensation	47,000	D-PLA (<2%)	PLA	[112]
		6.09		49.2	Molded with a twin extruder			PLA/Aspen wood particles 10%	[113]
		5.59		50.9	Molded with a twin extruder			PLA/Aspen wood particles 20%	[113]
		4.81		52.1	Molded with a twin extruder			PLA/Aspen wood particles 30%	[113]
		3.70		45.5	Molded with a twin extruder			PLA/Aspen wood particles 40%	[113]
		7.11		48.2	Molded with a twin extruder			PLA/Willow wood particles 10%	[113]
		6.15		49	Molded with a twin extruder			PLA/Aspen wood particles 20%	[113]
		5.08		47.2	Molded with a twin extruder			PLA/Aspen wood particles 30%	[113]
		4.26		44.1	Molded with a twin extruder			PLA/Aspen wood particles 40%	[113]
		4.6		37.5	Two-stage melt polycondensation	44,000	D-PLA (<2%)	PLA-PHS (95–5%)	[112]
		7.8		16.9	Two-stage melt polycondensation	33,000	D-PLA (<2%)	PLA-PHS (90–10%)	[112]
		15.3		22.5	Two-stage melt polycondensation	21,700	D-PLA (<2%)	PLA-PHS (80–20%)	[112]

## Data Availability

Not applicable.
